# Dr. Edward Brown-Sequard’s Experimental and Clinical Researches—Epilepsy

**Published:** 1857-08

**Authors:** 


### SELECTIONS.

### *Dr. Edward Brown-Sequard's Experimental and Clinical Researches, applied to Physiology and Pathology.*

EPILEPSY—(Continued).

§ XIV. We have tried, in the preceding part of this paper (sec. XIII.), to show the deficiencies of the principal theories of epilepsy. We will now state our own views; but, before doing so, we wish to declare that we do not pretend to give here a complete theory of epilepsy; we will merely try to elucidate some of the principal questions on this difficult subject.

I have ascertained upon my epileptic animals that the brain is not essential to the production of epileptiform convulsions. After I have taken away the brain proper, in one of these animals, I find that I can produce a fit almost as easily as before the operation, by pinching the skin of the face and neck. The only difference is, that the fit is not so violent, in consequence of the loss of blood. We find that still weaker convulsions may be caused by pinching the face and neck, if, besides the cerebral lobes, we take away the cerebellum, and even the whole of the basis of the encephalon, except the medulla oblongata and the pons Varolii.

From these experiments, it results that, in my animals, epilepsy has its seat in either the pons Varolii, the medulla oblongata, or the spinal cord, or in these three parts together. It is very probable that its seat is in the upper part of the spinal cord, in the medulla oblongata, and the pons Varolii, where the roots of the trigeminal and of the first spinal nerves have their origin. According to some experiments made by Edward Weber and Dr. R. B. Todd, the faculty of producing epileptiform convulsions does not belong to the spinal cord. E. Weber (Art. *Muskelbewegung*, p. 16, in Wagner's *Handworterbuch der Physiol.*) says, that the application of an electro-magnetic current to the spinal cord of frogs produces tetanic convulsions, while its application to the medulla oblongata causes alternate contractions and relaxations, as in epileptic fits. Dr. R. B. Todd (London Med. Gazette, May 11, 1849) states, that while the convulsions excited by the electro-magnetic current passing through the spinal cord and medulla oblongata are tetanic, the muscles being thrown into a state of *fixed* contraction, those which ensue when the current is transmitted through the region of the meso-cephalon and corpora quadrigemina are *epileptic*, being combined movements of *alternate* contraction and relaxation, flexion and extension, affecting the muscles of all the limbs, of the trunk, and of the eyes, which roll about just as in epilepsy. We have performed similar experiments upon rabbits and frogs, which have given almost the same results. In rabbits, when the current has passed through the pons Varolii and the tubercula quadrigemina, there were alternate movements of flexion and extension, resembling those of epilepsy, but much more extensive. When the current passed through the medulla oblongata, there were tetanic movements of the anterior limbs, with epileptiform convulsions of the posterior limbs; sometimes the anterior limbs also had epileptiform convulsions. When the current passed through the spinal cord, a tetanic spasm was produced. We have found that a state strongly resembling a fit of epilepsy exists after a transversal section of the upper part of the medulla oblongata, which state continues to exist as long as the animal lives. We must not, however, conclude from these experiments that the seat of epilepsy is only and always in one or in all of these parts—the tubercula quadrigemina, the pons Varolii and the medulla oblongata. Pressure upon these parts has often taken place in man without causing epileptiform convulsions, or convulsions of any kind. More than ten of the cases of organic diseases of the encephalon, collected by Abercrombie (*Path. and Pract. Researches on the Diseases of the Brain and Spinal Cord*, 4th ed., 1845, pp. 433–457), afford sufficient proof of this assertion. The results of the experiments of Weber, of Dr. Todd, and of our own, are certainly interesting, but they cannot lead to the conclusion that the convulsions of epilepsy in man result *constantly* from some affection of the quadrigeminal bodies (as Dr. Todd believes), or of the pons Varolii and medulla oblongata. It must be remembered that the experiments upon animals are made on healthy nervous centres, and that disease changes the vital properties of these centres. Tetanus, or at least tetanic convulsions, are sometimes due to diseases of the encephalon; and we have shown already (sec. X.) that the nature of the convulsions has not any constant relation with the parts of the cerebro-spinal axis (spinal cord or encephalon), primarily diseased in epilepsy. We know that the muscles animated by nerves arising from the encephalon, or by nerves from the spinal cord, very often exhibit the same kind of convulsions in epilepsy, in tetanus, in hydrophobia, in poisoning, etc. Besides, in a great many epileptics, the first convulsions in an attack are tonic (tetanic), and they are succeeded by clonic convulsions. In other epileptics the fits are sometimes entirely tetanic, and sometimes, though more rarely, entirely clonic. In certain animals, Dr. Martin-Magron and myself have discovered (see my *Experimental Researches Applied to Physiology and Pathology*, New York, 1853, p. 20) that irritation of the medulla oblongata caused by tearing out the facial nerve causes convulsions, which are partly tonic and partly clonic. Other irritations of the medulla oblongata, of the upper part of the spinal cord, of the pons Varolii and of its peduncles, of the tubercula quadrigemina, of the auditory nerve, etc., cause also tonic and clonic convulsions (see my work just quoted, pp. 18–23 and p. 99). These facts, and many others, compared to the effects of galvanization, show positively that different kinds of irritation produce different effects; and, therefore, we cannot conclude from the fact that epileptiform convulsions are produced by galvanic irritation of the pons Varolii, or other parts of the encephalon, that it is an irritation of these nervous centres which causes epilepsy in man.

If we neglect the nature of the convulsions, and take notice only of the parts of the body where they first occur, we arrive at the conclusion that the seat of epilepsy is very variable. Usually, however, the first spasmodic contractions occur in the muscles of the larynx, of the neck, of the eyes, of the chest, of the face, and in the blood-vessels of the brain proper, as we will show hereafter; and as these parts are animated by nerves coming from the encephalon and from the upper parts of the spinal cord, it seems that the seat of epilepsy is usually in some of these parts, if not in all. But the seat of this disease may be in other parts of the spinal cord, as seems to be proved by the production of the first spasmodic contractions in one of the limbs, either the inferior or superior. After the first fits, all the muscles of the body may be attacked with convulsions; so that, if we take notice of the loss of the actions of the brain proper, there is ground for thinking that the seat of the disease is both in those parts of the cerebro-spinal axis, where reside the faculties of perception and volition, and in those endowed with the reflex faculty; but this view is right only in appearance. We have shown already (sec. XIII.) that the loss of perception and volition does not prove that epilepsy has its seat in the brain proper; we will try, in a moment, to show the great probability that a contraction of the blood-vessels of the brain proper, due to an irritation of their nerves in the spinal cord and medulla oblongata, causes the loss of the cerebral faculties; and as regards the increase of the reflex faculty, we will show that a partial and a local increase is sufficient for the production of fits.

Are epileptic fits always the result of an excitation of the cerebro-spinal axis? We think that it is so; but we consider it possible, however, that the excitation arises from chemical and physical changes taking place in the elements of the nervous centres, from bad nutrition and other causes. In this case it is just the same thing as if an excitation was produced by a tumor, by a poison in the blood, or by a nervous influence arising from some irritated nerve, etc.

As physiology teaches that irritation of the simple direct motor side of the cerebro-spinal axis cannot cause general convulsions, we are entitled to consider as reflex the convulsive movements which result from direct excitations of the nervous centres, as well as those which result from irritations coming from peripheric nerve-fibres. The so called *centric* and *eccentric* causes of excitation of epileptic fits, both act on, or through the sensitive side of the cerebro-spinal centres, and consequently both act on the reflex faculty of these centres, so that they both ought to be called reflex excitations.

We think epilepsy depends in a great measure on an increased reflex excitability of certain parts of the cerebro-spinal axis. We shall no longer speak of reflex *faculty* or reflex *property*, because these words do not express what they mean. In all muscular and nervous tissues we find two distinct properties: a property of producing action, the force of which may vary extremely; and a property of receiving excitations, which we call excitability. One of these two properties may be very strong, while the other is very weak. Take, for instance, the muscles of cold-blooded animals; when the temperature is high, on the contrary, the least excitability induces them to contract, but their contraction is without force. Again, if we take an atrophied muscle, we find, sometimes, that it may be excited to contract by a galvanic current too weak to excite contractions in a healthy muscle, while, if we apply a strong stimulus to both, we find that the healthy muscle contracts with much more force than the atrophied one. Many experiments, which we will publish in another paper, have shown us that the reflex faculty of the cerebro-spinal axis is composed, as the muscular contractility is, of two elementary, vital properties, one of which we call the *reflex excitability*, and the other the *reflex force*. The cerebro-spinal axis may have a great reflex force, and very little excitability. It may, on the contrary, have an excessive reflex excitability with very little reflex force. In almost all epileptics, if not in all, the reflex excitability is increased, while the reflex force is rarely above, and often below its normal degree. The reflex excitability may not be much increased, although it is sufficient for the production of the fit, when certain excitations exist. I have found in my animals that there is not a great increase of the reflex excitability of the cerebro-spinal axis, except in a part of the the spinal cord which is separated from the rest, and has no share in the fits. In several persons attacked with epilepsy, I have ascertained that the excitations most capable of producing reflex movements did not act more powerfully than in healthy persons, although the experiments were made a short time before a seizure—that is, at a time when the reflex excitability ought to have been at its highest degree. In a young girl, particularly, we have ascertained that tickling the sole of the foot, the axilla, the lips, etc., produced less reflex movements than usual, although she was then expecting a fit, which came on, in fact, about ten minutes afterward. The researches made by Romberg and Professor Hasse (see his admirable work on *Krankheiten des Nervenapparates*, in Virchow's *Handbuch der Pathalogie*, Vol. IV., Part 1st, 1855, p. 254) on the production of reflex movements during fits of epilepsy, cannot prove much against or in favor of the existence of a great reflex excitability, or reflex force in epileptics, because if the experiment be made in the beginning of the fit, it is almost impossible to know whether the convulsions result from the experimental excitations, or are normal parts of the fit; and if the experiment is made at the end of the fit, the absence then of reflex movements proves only that the fit has exhausted the vital properties of the muscular and nervous tissues. Hasse concludes, from his own and from Romberg's experiments, that the greatest variety in the energy of reflex phenomena exists during the fits of epilepsy.

Whilst we admit that in epilepsy there is almost always, and perhaps always, an increased reflex excitability, alone or together, with an increased reflex force, we admit also that there is, in a great many cases of fits of epilepsy, a special kind of excitation, acting on the nervous centres. There are, therefore, three distinct elements for the production of a fit.

1st. Increase of the *force* of the reflex property.

2d. Increase of the *excitability* of this property.

3d. An excitation of a special nature, or a very violent one.

Of these three elements, the last two are the most frequent, and perhaps, as we have said, the first of these two is essential. As regards the share of special excitation in the causation of epilepsy, the cases we have related of the cure of this disease by the section of a nerve, by ligatures, etc., show how considerable it must be. But in my animals we have, in this respect, a better illustration. When the nerves going to the parts of the face and neck, by the irritation of which we are able to cause fits, are laid bare, we find that their irritation does not produce convulsions. If, in these animals, the fits depended only upon an increased reflex excitability of the parts of the nervous centre, whence the nerves originate, we should see convulsions follow when we irritate the trunks of these nerves. As there are none, we must admit that when an irritation (and a slight one is often sufficient) to the cutaneous ramifications of these nerves in the skin causes a fit, there is something special in the nature of the excitation springing from these cutaneous nerves. However, there is in my epileptic animals an increased degree of reflex excitability in the cerebro-spinal axis, as we find, even after the section of the nerves of the face and neck, that they have convulsions sooner, and lasting longer, than a healthy animal, when we prevent them from breathing for two or three minutes.

(To be Continued.)

